# Plasmid diversity of *Serratia marcescens* and *Klebsiella pneumoniae* isolates involved in two carbapenem-resistant *Enterobacteriaceae* outbreaks in a Swiss hospital

**DOI:** 10.1128/spectrum.03284-24

**Published:** 2025-05-21

**Authors:** Florian Mauffrey, Claire Bertelli, Gilbert Greub, Bruno Grandbastien, Laurence Senn, Dominique S. Blanc

**Affiliations:** 1 Infection Prevention and Control Unit, Infectious Diseases Service, Lausanne University Hospital and University of Lausanne 30635, Lausanne, Switzerland; 2 Institute of Microbiology, Lausanne University Hospital and University of Lausanne 30635, Lausanne, Switzerland; 3 Swiss National Reference Center for Emerging Antibiotic Resistance, Fribourg, Switzerland; Johns Hopkins University, Baltimore, Maryland, USA

**Keywords:** plasmids, outbreak, carbapenemase, typing, *Enterobacteriaceae*

## Abstract

This study investigates two distinct carbapenemase-producing *Enterobacteriaceae* outbreaks involving patients and contaminated sink traps at the University Hospital of Lausanne. It focuses on the diversity and transmission dynamics of plasmids carrying carbapenemase genes. Between 2022 and 2023, 57 carbapenem-resistant *Klebsiella pneumoniae* and *Serratia marcescens* isolates were collected and analyzed. Core-genome MLST confirmed genetic similarity among isolates, linking the outbreaks to sink trap contamination. DNA extraction, sequencing (MinION/Illumina MiSeq), and assembly were performed, followed by ARG screening and plasmid typing. Plasmids were annotated, clustered, and compared using core SNP distances and structural analyses. Known plasmids were identified through PLSDB database matching. Eight MLST types were identified in *K. pneumoniae* and one (ST356) in *S. marcescens*. Analysis of 52 *bla*-carrying plasmids revealed 22 plasmid clusters, including 6 *bla*_NDM-1_ clusters in *K. pneumoniae* and 4 *bla*_KPC-2_ clusters in *S. marcescens*. Plasmids showed close relatedness within and across patient and environmental isolates, with core SNP distances ranging from 0 to 18. Some *bla*_NDM-1_ plasmids in *K. pneumoniae* clustered tightly, suggesting persistence and potential cross-contamination routes. The findings highlight sink traps as critical reservoirs for carbapenem-resistant *Enterobacteriaceae* and plasmids, promoting resistance gene spread across species. The observed plasmid diversity indicates transmission can occur independently of bacterial clonal spread, challenging traditional outbreak definitions.

**IMPORTANCE:**

This research is critical in addressing the growing threat of antibiotic resistance, driven by the spread of resistance genes through plasmids. Plasmids, which can transfer between different bacteria, play a major role in spreading multidrug resistance, posing a serious challenge to healthcare systems worldwide. By highlighting how plasmids can move independently of bacterial spread, this study reveals the complexity of resistance transmission. It also underscores the importance of environmental reservoirs, such as hospital sink traps, in harboring and spreading resistant bacteria. These findings emphasize the need for better monitoring of plasmids and targeted infection control measures to prevent the spread of resistance genes and protect the effectiveness of current antibiotics.

## INTRODUCTION

The emergence and proliferation of carbapenem-resistant *Enterobacteriaceae* (CRE) represent a great challenge to public health, particularly within the hospital setting where vulnerable patient populations are at heightened risk (1). Carbapenems have long been regarded as last resort antibiotics for treating severe infections caused by multidrug-resistant gram-negative bacteria (2). However, the escalating occurrence of carbapenem resistance has compromised the efficacy of these critical therapeutics. The evolution of carbapenem resistance is primarily driven by the acquisition of carbapenemase genes, which encode enzymes capable of hydrolyzing carbapenem antibiotics, rendering them ineffective (1). Additionally, resistance can arise from modifications in membrane permeability and the upregulation of efflux pumps, further exacerbating the challenge of treatment. Nevertheless, the production of carbapenemase remains a more significant concern for infection prevention, and most of the published outbreaks consist of CPE outbreaks (1). The impact of CRE outbreaks in hospitals is profound, encompassing increased morbidity and mortality, prolonged hospital stays, and substantial economic burdens due to the need for enhanced infection control measures and the use of more expensive, and often more toxic, therapeutic alternatives (3–5).

Hospital settings are particularly prone to outbreaks of carbapenem-resistant organisms due to several factors including the high density of susceptible patients, frequent use of invasive devices, and intensive antibiotic pressure (6). The *Enterobacteriaceae* family, which includes clinically significant genera such as *Escherichia*, *Klebsiella*, and *Enterobacter*, has demonstrated a troubling propensity for developing and disseminating carbapenem resistance (7). Among these, *Klebsiella pneumoniae* are often implicated in hospital-associated infections and have been frequently reported to harbor carbapenemase genes such as *bla*_KPC_, *bla*_NDM_, *bla*_VIM_, and *bla*_OXA_ variants (8–10). *Serratia marcescens*, another member of the *Enterobacteriaceae* family, has gained attention as an emerging pathogen despite being less frequently implicated in outbreaks (11). *S. marcescens* outbreaks have been associated with various carbapenem-resistance genes, including *bla*_KPC-2_, *bla*_KPC-3_, and *bla*_OXA-48_. The dissemination of carbapenemase genes is frequently mediated by plasmids, which are mobile genetic elements capable of transferring resistance determinants between bacteria (12). They have been extensively described in *Enterobacteriaceae* (13). These plasmids often carry multiple resistance genes, enabling bacteria to withstand a wide range of antibiotics. The horizontal transfer of plasmids among *Enterobacteriaceae* facilitates rapid and widespread dissemination of resistance within and between bacterial species. Plasmids may also carry genes that enhance bacterial survival and virulence, further complicating treatment and control efforts (14, 15). Their ability to spread resistance genes across diverse bacterial populations underscores the critical need for stringent infection control and antimicrobial stewardship in hospital settings. The transmission dynamics of CRE are complex and multifactorial, involving patient-to-patient transmission, carbapenemase genes dissemination through plasmids transmission, environmental contamination, and lapses in infection control practices. Understanding the interplay between these factors is crucial for the development of effective prevention and control strategies.

The reservoirs of carbapenem-resistant bacteria within hospital environments extend beyond infected or colonized patients. Environmental surfaces, including sinks, drains, and other wet surfaces, have been identified as significant reservoirs of CRE (16, 17). These sites provide a conducive environment for the persistence and transmission of these organisms, facilitated by biofilm formation which enhances microbial survival and resistance to disinfectants. Contaminated sink traps, in particular, have been implicated in several nosocomial outbreaks, serving as a nexus for the transfer of resistant bacteria from the environment to patients, often via the hands of healthcare workers or through direct contact with contaminated surfaces (18–20).

In this study, we extended the analysis of two different outbreaks of carbapenemase-producing *Enterobacteriaceae* involving patients and contaminated sink traps at the University Hospital of Lausanne. We isolated and clustered the different plasmids involved in these outbreaks in order to get more insights about the plasmids diversity and dynamics involved in these outbreaks.

## MATERIALS AND METHODS

### Sample selection

Between 2022 and 2023, two outbreaks of carbapenem-resistant *K. pneumoniae* (21) and *S. marcescens* were suspected in two different wards at the University Hospital of Lausanne. The presence of carbapenem-resistance genes in these isolates was determined using the lateral flow immunoassay NG-Test CARBA-5 (NG Biotech, France) and was confirmed by the national center for emergent antibiotic resistances (NARA, Fribourg, Switzerland).

The first outbreak involved *bla*_NDM-1_ carrying *K. pneumoniae* isolates from five patients and sink traps located in a neurosurgical intermediate care unit and has been described elsewhere (21). The second potential outbreak involved *bla*_KPC-2_ carrying *S. marcescens* isolates. Following an investigation of sink traps in adult ICUs, this resistant pathogen was recovered from two patients hospitalized 17 months apart in these ICUs. The retained hypothesis was that the first patient was at the origin of sink traps contamination and the second patient was contaminated by the sink traps. In both outbreaks, the high similarity of bacterial genomes was assessed by core genome MLST (cgMLST). Other isolates carrying *bla*_NDM-1_ (six *K*. *pneumoniae*, four *Klebsiella oxytoca*, three *Escherichia coli*, two *Pseudomonas aeruginosa*, two *Acinetobacter gr. baumannii*, one *Citrobacter freundii*, and one *Enterobacter cloacae*) and *bla*_KPC-2_ genes (eight *K*. *pneumoniae*, three *E. coli*, two *E. cloacae*, one *K*. *oxytoca*, one *C*. *freundii*, and one *Morganella morganii*) recovered from hospitalized patients were included in the study in order to capture the most representative diversity of plasmids in our hospital. In total, 57 isolates were included in this study. Detailed information on these isolates is provided in Table S1.

### Long read genome sequencing and assembly

DNA was extracted from the 64 isolates using the Promega Wizard DNA Purification Kit according to the manufacturer’s instructions and quantified using the Qubit fluorometer. Genome sequencing was performed on a MinION Mk1C device using R10.4.1 flow cells and the Native Barcoding Kit 24 V14. Ten to twelve samples were multiplexed on each flow cell, and sequencing was carried out for 72 h. After sequencing, the final raw reads were basecalled using the super accurate dna_r10.4.1_e8.2_400bps_sup@v4.1.0 model with Dorado v0.3.2. Reads files were filtered with nanofilt v2.8.0, and quality was checked with nanoq v0.10.0 (22). Genomes were assembled with Flye v2.9.1, and assemblies were further polished with Medaka v1.8.2 and Homopolish v0.4.1 (23, 24). *S. marcescens* isolates were also sequenced on an Illumina MiSeq platform. Genomes assembly from short reads was performed with Unicycler inside Bionumerics v8.1.1 (bioMérieux, Applied Maths NV, St Martens Latem, Belgium). *bla*_NMD-1_ carrying *K. pneumoniae* and *bla*_KPC-2_ carrying *S. marcescens* isolates were typed using a cgMLST approach. For *S. marcescens* isolates, short reads assemblies were used instead of long reads assemblies. cgMLST profiles were calculated using pyMLST v2.1.3 with the corresponding Ridom scheme (25–27). Minimum spanning trees (MSTs) were generated from these profiles using Grapetree v1.5.0 with an MSTreeV2 matrix type (28). Isolates MLST was determined using pubMLST (29).

### Assembly analysis

**Fig 1:**
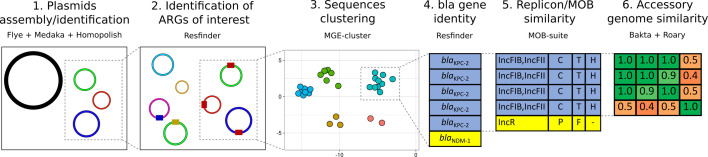
Plasmid typing workflow. The tools used at each step are shown.

For each assembly, contigs were classified as either chromosomes (size >1 Mbp) or putative plasmids. Antimicrobial resistance genes (ARGs) were screened in all contigs using ResFinder v4.1.11 (30). Plasmid contigs were typed with MOB-suite v3.1.4 (31). To cluster plasmid contigs based on sequence similarity, MGE-cluster v1.1.0 was used, testing perplexity values of 10, 15, 20, and 25 with a minimum cluster size of 2 due to the small size of our collection (32). Optimal results were obtained with a value of 10, which tended to better preserve local structures (data not shown). Plasmid contigs were annotated using Bakta v1.9.3, and the pangenome was calculated for each plasmid family using Roary v3.13.0 (33, 34). Roary genome profiles were used to calculate accessory genome similarities using the Jaccard index (R, vegan v2.6-6). For plasmid sequences belonging to outbreak plasmid clusters, core SNP distance was calculated using snp-dists v0.8.2 (https://github.com/tseemann/snp-dists) from the core genes alignment generated by Roary. The full plasmid typing workflow is described in Fig. 1.

To associate known plasmids with those identified in this study, the PLSDB database was downloaded (https://ccb-microbe.cs.uni-saarland.de/plsdb). The mash distances between all plasmids identified in this study and those from the PLSDB database were calculated using mash v2.3-7 (35). Plasmid from the PLSDB database with the minimum mash distance was defined as the most similar sequence. Plasmid structures were compared using annotation files generated by Bakta into BRIG (BLAST Ring Image Generator) v.0.95 (36).

## RESULTS

### Isolate genotyping

**Fig 2:**
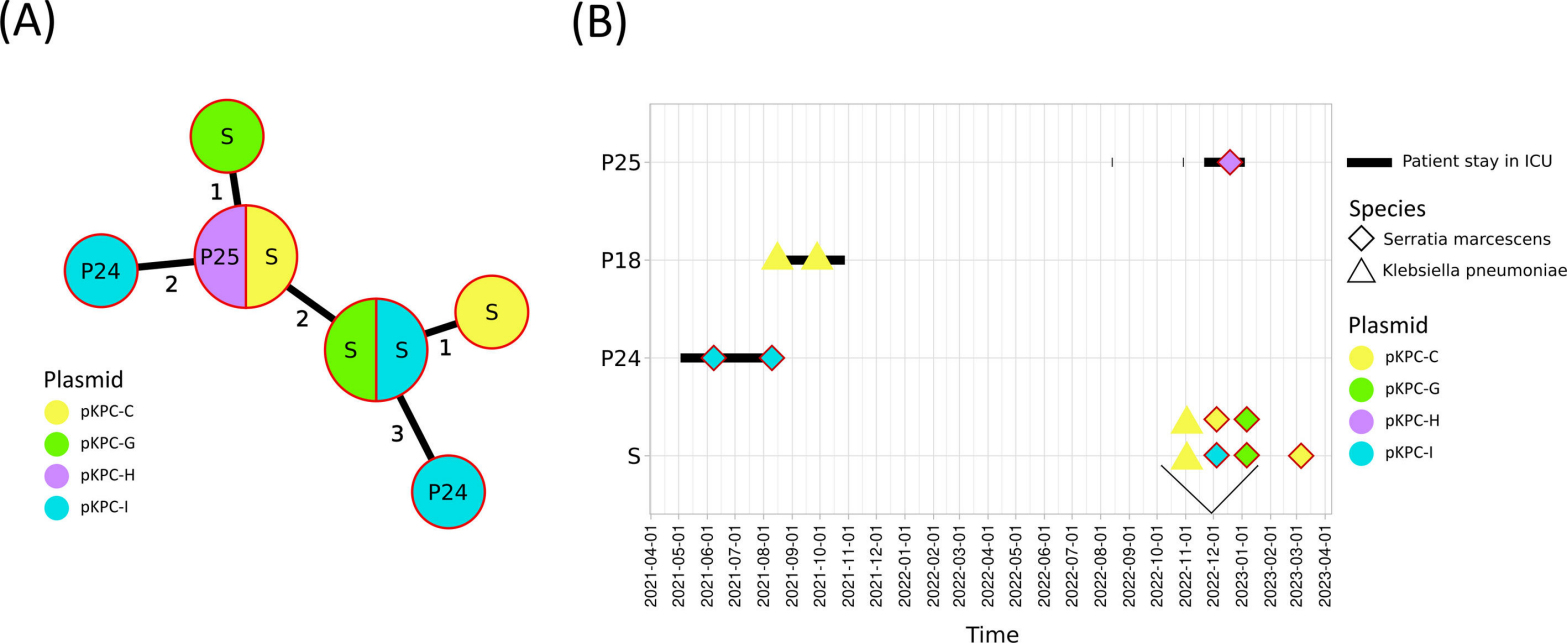
Genomic and epidemiological data of *Serratia marcescens* isolates carrying the *bla*KPC-2 gene recovered from patients (P) or from sink traps (S) from the same unit where patients were hospitalized. Colors indicate the plasmid carrying the *bla*KPC-2 gene in each isolate. (**A**) Minimum spanning tree (MST) based on the cgMLST profile of all isolates. The loci distance between each isolate is indicated. (**B**) Hospital stays of patients colonized or infected with *bla_KPC-2_ S. marcescens* or *K. pneumoniae* (black lines). Sampling dates are represented by a diamond (for *S. marcescens* isolates) or a triangle (for *K. pneumoniae* isolates). A red outline indicates they belonged to the genomic cluster illustrated in panel A.

**Fig 3:**
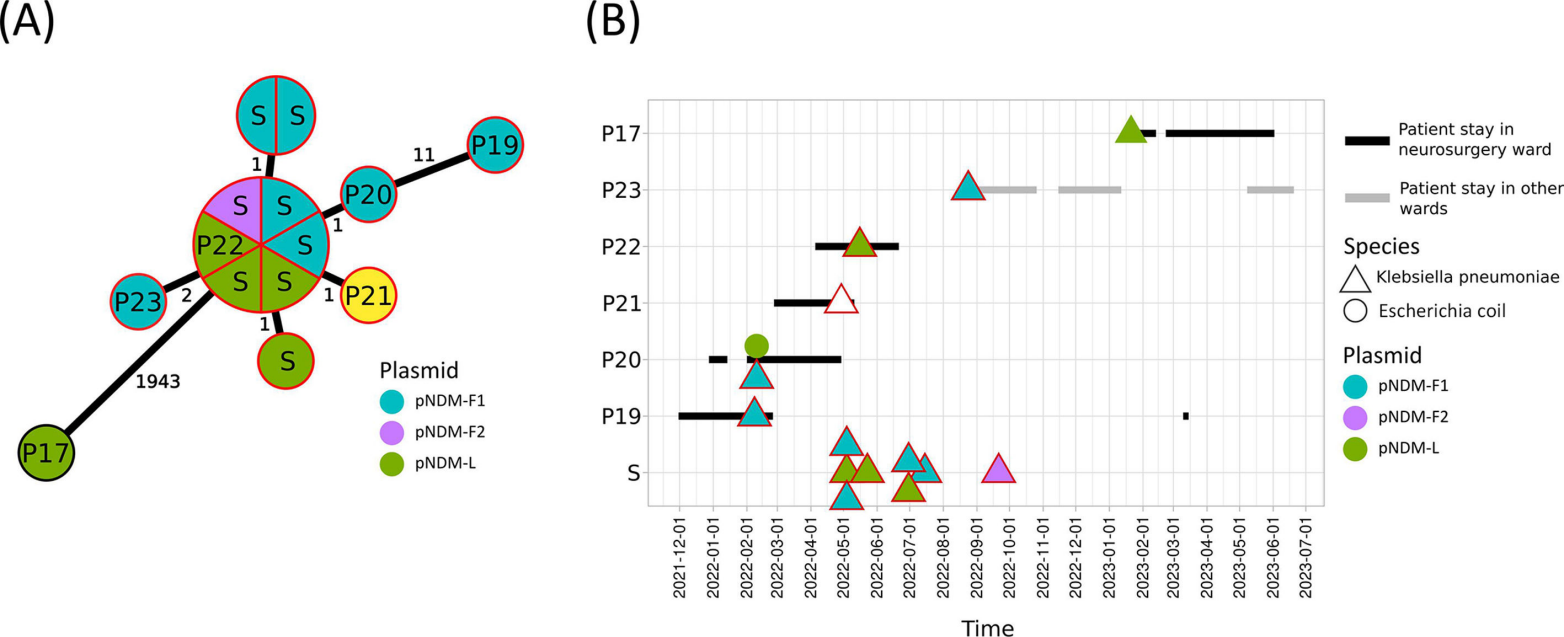
Genomic and epidemiological data of isolates carrying the *bla_NDM-1_* gene recovered from patients (P) or from sink traps (S) from the same unit where patients were hospitalized. Colors indicate the genomic location of the *bla_NDM-1_* gene in each isolate. (**A**) Minimum spanning tree based on the cgMLST profile of all *K. pneumoniae* isolates. The loci distance between each isolate is indicated. (**B**) Hospital stays of patients colonized or infected with *bla_NDM-1_ K. pneumoniae* or *E. coli* (black and gray lines). Sampling dates are represented by a triangle (for *K. pneumoniae* isolates) or a circle (for *E. coli* isolates). A red outline indicates they belonged to the genomic cluster illustrated in panel A.

Short-read assemblies were used for MLST and cgMLST analyses of the eight *S*. *marcescens* isolates. All belonged to ST356 (Table S1) and formed a very tight cluster in the MST with a maximum observed distance of three loci (Fig. 2A). Long-read assemblies were used for MLST and cgMLST analyses of *K. pneumoniae* isolates. Eight different MLST STs were identified: ST11, ST147, ST258, ST268, ST39, ST392, ST4834, and ST584 (Table S1). A cluster of highly related isolates (0–11 loci difference) was observed within ST268 (Fig. 3A). This cluster comprised 13 isolates from five patients and multiple sink traps. The epidemiology of this cluster has been thoroughly described elsewhere (21).

### Plasmid identification

For 54 out of 57 isolates, long-read assemblies were constituted of a well-defined chromosome and putative plasmids (sequences of 6,444 to 399,384 kb) with either *bla*_NDM-1_ or *bla*_KPC-2_ genes (Table S2). No putative plasmid was detected in isolate P06-37368, and the *bla*_NDM-1_ gene was located on the chromosome. The expected *bla*_NDM-1_ gene was not found in isolate P04-35748, although sequencing quality was equivalent to other samples. Although putative plasmids were present in the P21-39353 assembly, the *bla*_NDM-1_ gene was found only on the chromosome. Only sequences with *bla*_NDM-1_ or *bla*_KPC-2_ genes were selected for further analysis, representing a total of 54 sequences. Among them, two sequences lacked a replicon sequence and were discarded. Thus, a total of 52 plasmid sequences were used for further analysis.

### Plasmid typing

These 52 plasmids were grouped using MGE-cluster (Fig. S1, minimum membership probability >0.3), *bla* gene types and replicon/MOB gene types (see Materials and Methods). The resulting clusters grouped plasmids with a common backbone and a high overall sequence similarity. In order to consider plasmids plasticity, subclusters within each cluster were further defined based on accessory genome similarities, using an empirical similarity threshold of 0.7 (Table S3). In total, 22 different plasmid clusters were defined, among which 13 were composed of only one representative. Characteristics of plasmid clusters related to the two outbreaks are summarized in Table 1.

**TABLE 1:** Plasmid characteristics from isolates linked to the two outbreaks^*a*^

Plasmid cluster	Mean length (bp)	Carbapenem resistance gene	Replicon	Relaxase	mpf	orit	Predicted mobility	Isolates
pKPC-C	48,517	*bla*_KPC-2_	IncFIB, (IncFII), rep_cluster_2183	-^*b*^	-	-	Non-mobilizable	P18, S2
pKPC-G	64,753	*bla*_KPC-2_	IncFIB, IncFII, rep_cluster_2183	F,F	T	-	Conjugative	S2
pKPC-H	223,156	*bla*_KPC-2_	IncFIB, IncFIB, IncFII, IncFII, rep_cluster_2183	F,F	F	-	Conjugative	P25
pKPC-I	99,775	*bla*_KPC-2_	IncFIB, IncFII, rep_cluster_2183	F,F	F	-	Conjugative	P24, S2
pNDM-F1	249,760	*bla*_NDM-1_	IncC, IncFIA, IncR, rep_cluster_1254	H,H	F	F,H	Conjugative	S1, P19, P20, P23
pNDM-F2	399,384	*bla*_NDM-1_	IncC, IncFIA, IncFIB, IncHI1B, IncR, rep_cluster_1254	H,H	F	F,H	Conjugative	S1
pNDM-L	173,391	*bla*_NDM-1_	IncC, rep_cluster_1254	H,H	F	H	Conjugative	S1, P17, P20, P22

^*a*^ Detailed information can be found in Table S3.

^*b*^ -, absence of the sequence.

### *S. marcescens* outbreak

Four different *bla*_KPC-2_ plasmid clusters were identified in *S. marcescens* isolates: pKPC-C, pKPC-G, pKPC-H, and pKPC-I (Fig. 2). Plasmids belonging to these four clusters had IncFIB, IncFII, and rep_cluster_2183 replicon sequences, except for pKPC-C-S2-40035 and pKPC-C-S2-40391 lacking the IncFII replicon sequence. The pKPC-C cluster included small plasmids (46–53 kb) classified as non-mobilizable (Fig. S2). All pKPC-C sequences were similar (mash distance < 0.02) to the pKPC_4_29_19 plasmid isolated in a *Salmonella enterica* strain in Italy in 2018, except it carried a *bla*_KPC-3_ gene (37). A pKPC-C plasmid was also found in *K. pneumoniae* isolates present both in sink traps (S2) and in isolates of patient P18. Despite carrying the same plasmid, patient and sink trap isolates were genetically different (Table S4). The two plasmids belonging to the pKPC-G cluster were exclusive to *S. marcescens* recovered from sink traps. These conjugative plasmids had an almost identical sequence of 64.7 kb (Fig. S3). pKPC-I plasmids were found in the three *S. marcescens* isolates from patient P24 as well as one *S. marcescens* isolate from a sink trap. These four plasmid sequences were also almost identical (99.7 kb) and classified as conjugative (Fig. S4). Both pKPC-G and pKPC-I plasmids were highly similar (mash distance <0.02 and <0.001, respectively) to pKPC-2 recovered from *K. pneumoniae* isolates involved in an outbreak in Germany (38).

While the P25 isolate was genetically closely related to all other *S. marcescens* isolates, its plasmid belonged to the pKPC-H cluster, which has never been encountered in other isolates from our collection. Interestingly, a similar *bla*_KPC-3_ plasmid (mash distance < 0.02), p1-LC-1302-2020-KPC3, was isolated from the *E. coli* strain Ecol_517 in Italy (39). Both pKPC-C and pKPC-I plasmid sequences had low core SNP distances ranging from 0 to 18 SNPs (Table S5).

### *K. pneumoniae* outbreak

Six different *bla*_NDM-1_ plasmid clusters were identified in *K. pneumoniae* isolates from patients and sink traps, with two of them involved in the *K. pneumoniae* outbreak: pNDM-F and pNDM-L (Fig. 3). pNDM-F was subdivided into two subclusters F1 and F2. pNDM-F1 plasmids were detected in isolates from three patients (P19, P20, and P23) and from sink traps. This cluster was composed of highly similar conjugative plasmids (~250 kb) with multiple replicon sequences: IncC, IncFIA, IncR, and rep_cluster_1254 (Fig. S6). Their closest reference in PLSDB was the p3347558_1 plasmid (Table S6). pNDM-F2 plasmid was only detected in *K. pneumoniae* isolated from a sink trap. pNDM-L plasmids were found in isolates from patients P17, P22, and from sink traps, although the isolate from patient P17 was genetically distant from the other *K. pneumoniae* isolates (Fig. 3A). A pNDM-L plasmid was also found in an *E. coli* isolate from patient P20. The pNDM-L cluster was composed of medium-sized conjugative plasmids (169–203 kb) with IncC and rep_cluster_1254 replicon sequences (Fig. S5). These plasmids were highly similar (mash distance < 0.001) to p3347558_1 and p33477089II_1 isolated from an *E. coli* and a *Citrobacter sedlakii*, respectively (40). Of notice, pNDM-L-S1-39377, pNDM-L-S1-39425, and pNDM-L-P22-39408 (169 kb) were nearly identical to p3347558_1 with mash distances < 0.0001 (Table S6). Both pNDM-F1 and pNDM-L plasmid sequences had low core SNP distances ranging from 0 to 14 SNPs, except for pNDM-L-P17-40226, which had a distance of 105–113 SNPs with other pNDM-L plasmids (Table S5). For patient P21 isolate, the *bla*_NDM-1_ gene was located on the chromosome, resulting from the integration of a pNDM-L plasmid (data not shown).

## DISCUSSION

In this study, we reported two outbreaks of CRE that occurred between 2022 and 2023 at the University Hospital of Lausanne. Given that carbapenemase genes are usually carried by plasmids, we investigated the plasmid diversity involved in these outbreaks. Unlike genome typing, plasmid typing is challenging due to its mosaic nature (41). Various approaches have been developed showing different levels of discrimination (42, 43). In this study, we combined different existing methods (MGE-cluster, mash distance, MOB-suite, and genome similarity) to achieve a high level of discrimination for epidemiological investigations.

The first outbreak involved *S. marcescens* carrying *bla*_KPC-2_, isolated from patients hospitalized in the ICU and from sink traps. The presence of carbapenemase genes in *S. marcescens* isolates has been reported sporadically over the past few decades, with variants such as VIM, IMP, OXA, and KPC being the most common (11). While most *S. marcescens* infections are individual cases, outbreaks have been reported, and nosocomial infections frequently occur in ICU and neonatology units, with isolates from various sources, highlighting *S. marcescens*' capability to survive in different environments (44–48). Patient-to-sink and sink-to-patient transmissions of *S. marcescens* have been reported in many studies (49–52). In our study, *S. marcescens* ST356 isolates were recovered from two ICU patients as well as from sink traps in this unit. All isolates were genetically highly similar based on cgMLST, suggesting a recent chain of transmission. However, the two patients were hospitalized 16 months apart, making direct transmission between them impossible. Initially, we hypothesized that the first patient, P24, contaminated the sink traps during hospitalization, and that P25 was subsequently exposed to these contaminated sink traps 16 months later. However, plasmid typing reveals a different story. The plasmid pKPC-I was present in P24 isolates, whereas P25 harbored pKPC-H, contradicting a clonal transmission of the resistance gene. In the sink traps, we identified the same *S. marcescens* genotype as in P24, but with three different plasmids: pKPC-I, similar to the one in P24 isolates; pKPC-G, unique to sink trap isolates; and pKPC-C, which was also found in *K. pneumoniae* from both the sink traps and patient P18. These findings highlight the limitations of our investigation, as we could not determine the origin of the pKPC-H plasmid in P25 or explain the presence of pKPC-G in the sink traps. Nevertheless, our results suggest that plasmid exchange likely occurred within the sink traps. As shown in previous research, this environment may serve as a reservoir for plasmids across various bacterial species, supported by the presence of pKPC-C in both *K. pneumoniae* and *S. marcescens* isolates from the sink traps (53, 54). pKPC-C plasmids were predicted as non-mobilizable, suggesting that transfer processes involving helper plasmids or phages likely occur in such environments.

All these plasmids had a multireplicon with IncF replicons (IncFIB and IncFII) along with rep_cluster_2183, which appears to be common among IncF plasmids (55). Although IncFIB/IncFII plasmids have been reported in *S. marcescens*, they are not commonly found in this species (56). Plasmids belonging to these four clusters had shared a common backbone similar to pQil plasmids commonly found in *S. marcescens*, suggesting that they could have derived from a common plasmid sequence. These plasmids were also characterized by the absence of other ARGs, correlating with their small sizes. IncF plasmids are the most described plasmid family, carrying various carbapenemase genes and are specific to *Enterobacteriaceae* (13). *bla*_KPC_ has been mostly found on IncF plasmids in *K. pneumoniae* responsible for the dissemination of this resistance in some countries (57–59). *bla*_KPC_ incF-like plasmids were rarely reported in *S. marcescens* strains and are usually detected in *K. pneumoniae* and *E. coli* (60, 61).

The second outbreak involved *bla*_NDM-1_ *K. pneumoniae* isolated from patients and sink traps in a neurosurgical intermediate care unit. While carbapenem-resistant *K. pneumoniae* mostly carried *bla*_KPC-2_ in reported outbreaks, the emergence of *bla*_NDM-1_ in this species has been described since 2009 in different locations including Europe (62). Similarly to *S. marcescens*, carbapenem-resistant *K. pneumoniae* has also been isolated from sinks as reported in different outbreaks (16). In our study, the *K. pneumoniae* clone ST268 was identified in five patients and various sink traps. All these isolates were highly similar by cgMLST. Isolates from patients P19 and P20 were collected prior to those from the sink traps, indicating that the sink traps may have been contaminated by these patients. This contamination could have subsequently led to the infection of other patients through contact with the sink traps. Patient P23 might also be part of the outbreak, as its isolate was genetically very similar to the others, even though this patient was in a different ward, suggesting that the clone may have spread to another part of the hospital. However, no additional isolates from this cluster were recovered.

The two plasmid clusters pNDM-F and pNDM-L were found among *K. pneumoniae* isolates belonging to the outbreak clone, present in both patients and sink traps. Patient P20 also carried an *E. coli* strain carrying a pNDM-L plasmid (pNDM-L-P20-39095), but only pNDM-F1 was detected in *K. pneumoniae* isolates from patients P19 and P20, suggesting that pNDM-L plasmids were brought to the sink traps via this *E. coli* strain. The low SNP distance between pNDM-L-P20-39095 and other pNDM-L plasmids further supports this idea. Again, this highlights the sink traps as a hotspot for plasmid exchange between different species. However, no *E. coli* carrying pNDM-L was found in these sink traps. Patient P17 was screened upon admission and tested positive for *K. pneumoniae* NDM. This indicates that he did not acquire the strain during his stay in our hospital, which was further confirmed by the significant genetic distance between the isolate and the outbreak clone. Notably, this isolate also carried a pNDM-L plasmid similar to those found in the outbreak strains. However, the high SNP distance from other pNDM-L plasmids suggests that pNDM-L-P17-40226 was not part of the hospital plasmid transmission chain. Finally, several *bla*_NDM-1_-carrying plasmids were found in *K. pneumoniae* isolates unrelated to this outbreak and were all different from the outbreak plasmids, with various replicon types, further highlighting the diversity of these plasmids within the hospital.

In addition to *bla*_NDM-1_, pNDM-F and pNDM-L plasmids contained multiple antibiotic resistance genes (Table S7) spanning various classes, a finding consistent with the well-documented tendency of *bla*_NDM_-carrying *K. pneumoniae* strains to harbor additional resistance genes (63, 64). Plasmids from both clusters had at least IncC and rep_cluster_1254 replicon sequences, with pNDM-F plasmids having additional IncFIA, IncF1B, IncHI1B, or IncR replicon sequences. IncC plasmids are widely distributed in gram-negative bacteria with a broad range of hosts, including *K. pneumoniae*, and have been associated with the dissemination of the *bla*_NDM_ carbapenemase gene (65, 66). Moreover, IncC and IncF are among the most frequently occurring replicon types of *bla*_NDM_ plasmids identified in *K. pneumoniae* (67). Interestingly, plasmids with sequences highly similar to pNDM-F1 plasmids were found at the University Hospital of Bern in an *E. coli* isolated from a patient repatriated from Macedonia (40). In their study, the authors showed that very similar *bla*_NDM-1_ plasmids have been reported in several countries around the globe, suggesting a global distribution of such *bla*_NDM-1_ IncC plasmid. Furthermore, this plasmid was also found in *K. pneumoniae* and *E. coli* isolates at the University Hospitals of Geneva, but none of the affected patients stayed in both hospitals, suggesting that the plasmid may also be spread in Switzerland (Diego Andrei, personal communication).

### Conclusion

In this study, we investigated two distinct outbreaks of CRE in a hospital setting, involving both patients and sink traps. We focused on characterizing the *bla*-carrying plasmids recovered from these isolates to better understand the dynamics of resistance gene dissemination. Our findings strongly reinforce the role of sinks as persistent reservoirs and sources of contamination, as highlighted by previous research (50). The sink traps demonstrated a remarkable diversity in bacterial species, carbapenemase genes, and associated plasmids. This diversity was particularly striking in the context of plasmid profiles, which appeared more varied than the genetic profile of the outbreak strains themselves. Specifically, within each outbreak clone, we identified two to three distinct plasmid clusters, indicating that plasmid diversification outpaced the diversification of their bacterial hosts.

Moreover, we observed that the plasmids involved in the outbreaks were not confined to a single species or outbreak scenario. Instead, they were also present in isolates from different species and in isolates unrelated to the documented outbreaks. This finding challenges the traditional boundaries used to define outbreak limits, as it suggests that plasmid transmission may extend beyond the immediate epidemiological cluster. In such scenarios, where carbapenem resistance is a concern, it becomes essential to consider plasmid circulation as a parallel and equally important factor alongside clonal spread. Plasmid mobility and their ability to cross species barriers mean that an outbreak cannot be fully understood or contained merely by tracking the bacterial clones. To accurately delineate the extent of an outbreak, both the spread of resistant clones and the circulation of resistance-carrying plasmids must be thoroughly investigated. This integrated approach could lead to more effective strategies for outbreak management and infection control, emphasizing the need for comprehensive surveillance of both bacterial strains and their associated plasmids.

## Data Availability

Raw sequencing data and plasmid sequences were deposited in the European Nucleotide Archive (ENA) under the Bioproject ERP161852. All accession numbers are detailed in Tables S1 and S2.

## SUPPLEMENTAL MATERIAL

Supplemental figures spectrum.03284-24-s0001.pdf Fig. S1 to S6.

Supplemental tables spectrum.03284-24-s0002.xlsx Tables S1 to S7.

ASM does not own the copyrights to Supplemental Material that may be linked to, or accessed through, an article. The authors have granted ASM a non-exclusive, world-wide license to publish the Supplemental Material files. Please contact the corresponding author directly for reuse.
