# Supplementary material for: Plasmid diversity of Serratia marcescens and Klebsiella pneumoniae isolates involved in two carbapenem-resistant Enterobacteriaceae outbreaks in a Swiss hospital
Source: Microbiol Spectr. 2025 May 21;13(7):e03284-24. doi: 10.1128/spectrum.03284-24 (PMC12210979; doi:10.1128/spectrum.03284-24)
Supplement: Supplemental figures — Fig. S1 to S6. [file spectrum.03284-24-s0001.pdf]

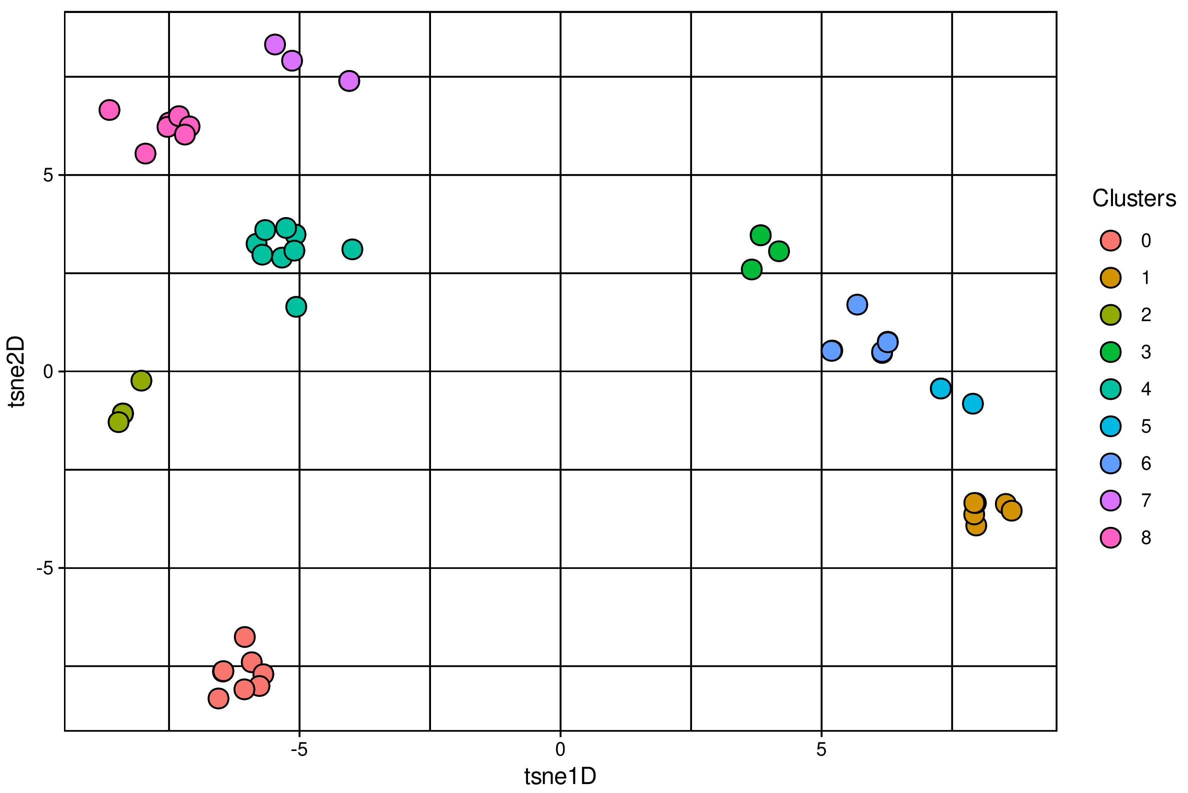

Figure S1: Plasmids clusters as defined by the MGE-cluster analysis. Plasmids belonging to no cluster were identified as cluster -1.

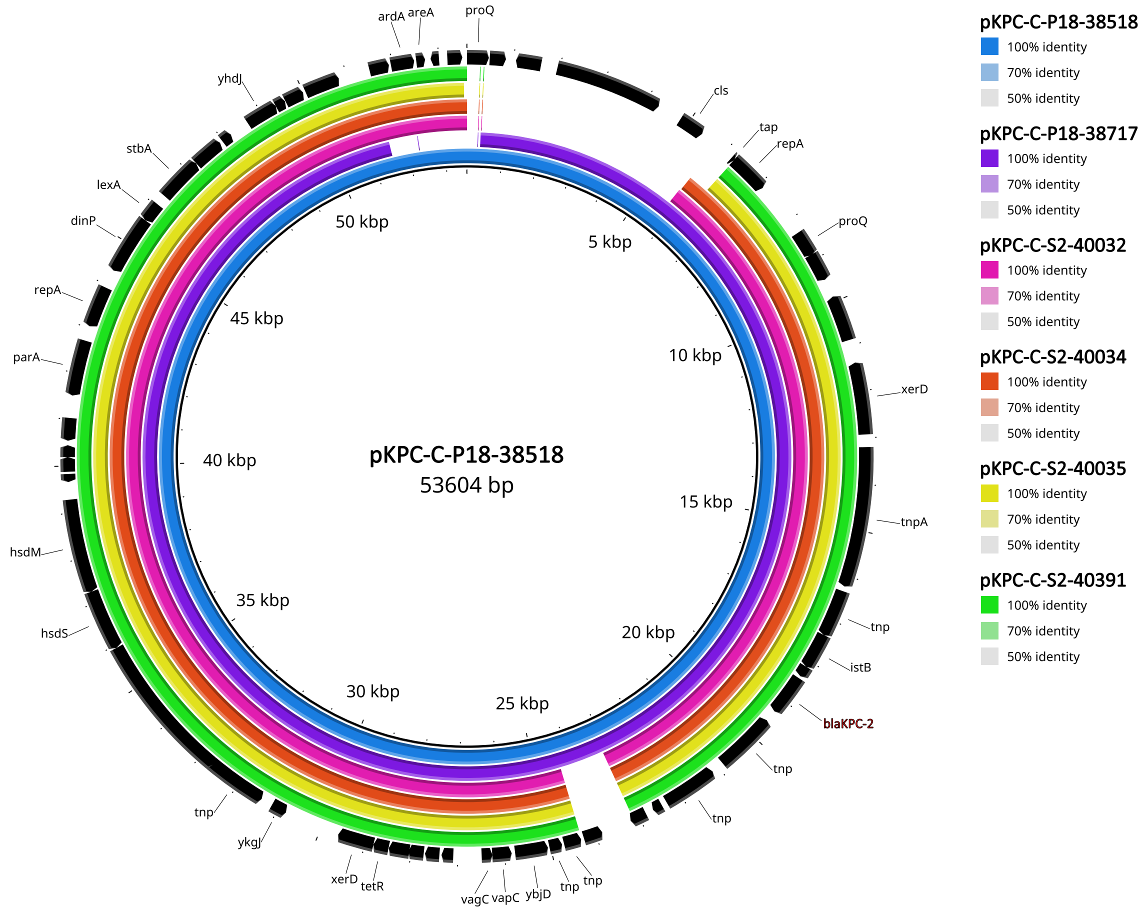

Figure S2: BLASTn comparison of the six pKPC-C plasmids sequences. Plasmid sequence pKPC-C-P18-38518 was used as reference sequence. The colored rings represent similarities to the reference sequence. CDS are depicted as black arrows in the outermost circle and $bla_{\text{KPC-2}}$ is represented in red. BRIG v0.95 was used for generating the figure.

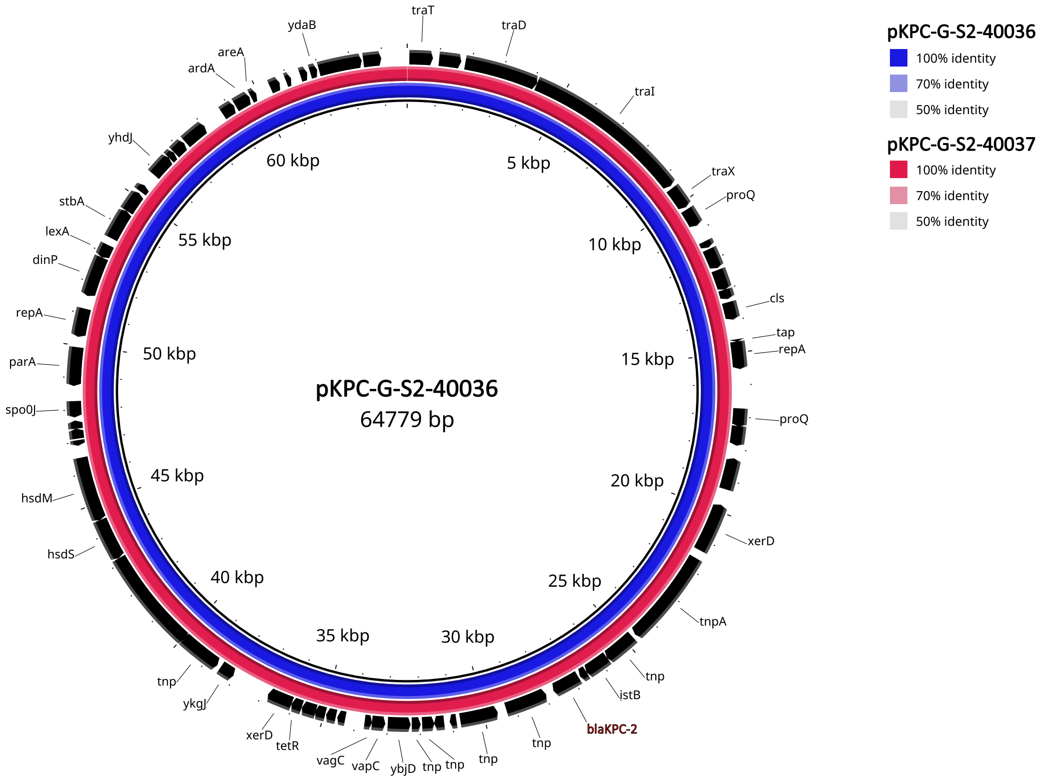

Figure S3: BLASTn comparison of the two pKPC-G plasmids sequences. Plasmid sequence pKPC-G-S2-40036 was used as reference sequence. The colored rings represent similarities to the reference sequence. CDS are depicted as black arrows in the outermost circle and *bla*$_{KPC-2}$ is represented in red. BRIG v0.95 was used for generating the figure.

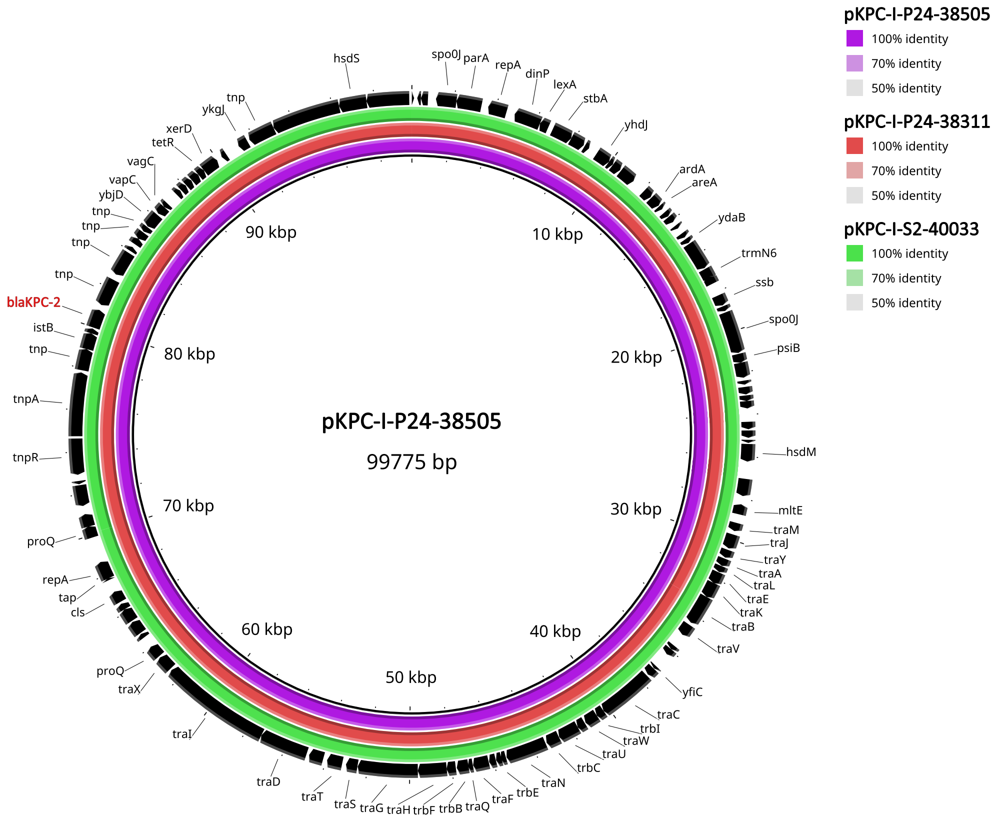

Figure S4: BLASTn comparison of the four pKPC-I plasmids sequences. Plasmid sequence pKPC-I-P24-38385 was used as reference sequence. The colored rings represent similarities to the reference sequence. CDS are depicted as black arrows in the outermost circle and *bla*$_{KPC-2}$ is represented in red. BRIG v0.95 was used for generating the figure.

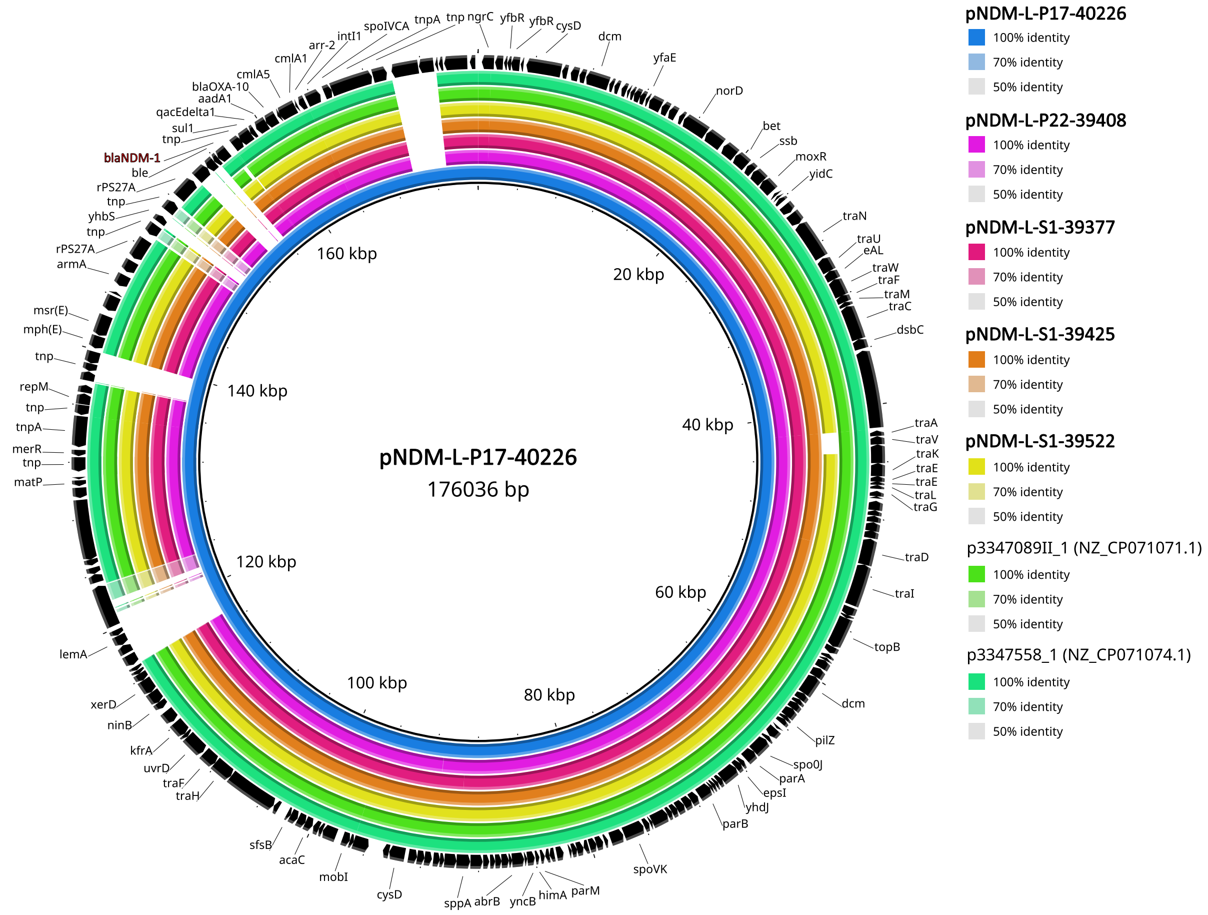

Figure S5: BLASTn comparison of the five pNDM-L plasmids sequences and the two very close plasmids found in Bern. Plasmid sequence pNDM-L-P17-40226 was used as reference sequence. The colored rings represent similarities to the reference sequence. CDS are depicted as black arrows in the outermost circle and *bla*$_{NDM-1}$ is represented in red. BRIG v0.95 was used for generating the figure.

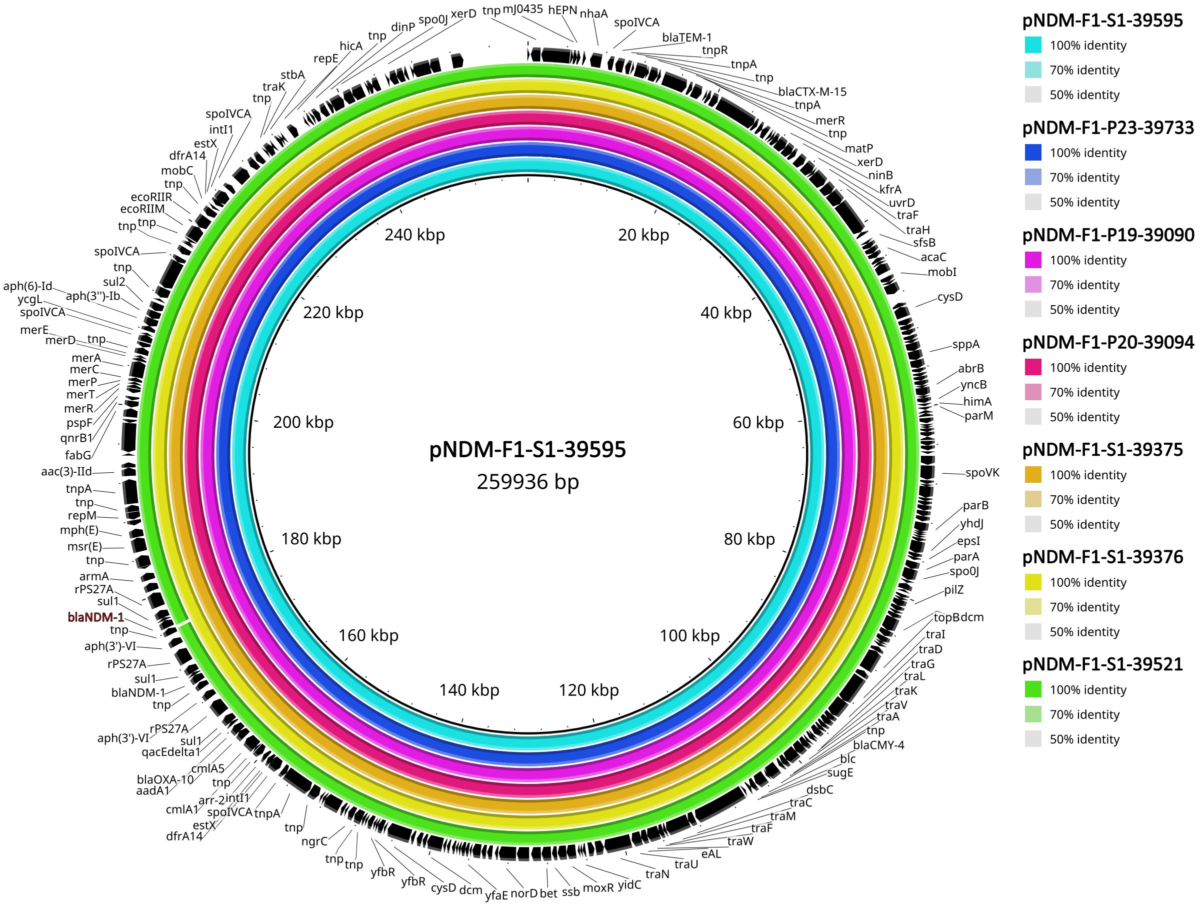

Figure S6: BLASTn comparison of the seven pNDM-F1 plasmids sequences. Plasmid sequence pNDM-F1-S1-39595 was used as reference sequence. The colored rings represent similarities to the reference sequence. CDS are depicted as black arrows in the outermost circle and *bla*$_{NDM-1}$ is represented in red. BRIG v0.95 was used for generating the figure.
